# Corticosteroid treatment in severe patients with SARS-CoV-2 and chronic HBV co-infection: a retrospective multicenter study

**DOI:** 10.1186/s12879-022-07882-6

**Published:** 2022-11-28

**Authors:** Mei Meng, Yufeng Chu, Sheng Zhang, Xuechuan Li, Jing Sha, Peng Wang, Yunliang Cui, Meihong Han, Xuan Dong, Wenqing Sun, Zhongfa Zhang, Yunxin Deng, Tao Wang, Djillali Annane, Shouqiang Jia, Dechang Chen

**Affiliations:** 1 Department of Critical Care Medicine, Ruijin Hospital, Shanghai Jiao Tong University School of Medicine, No 197, Rui Jin 2nd Road, Shanghai, 200025 China; 2 Department of Critical Care Medicine, The Second Hospital, Cheeloo College of Medicine, Shandong University, Jinan, Shandong China; 3 Department of Burn, Ruijin Hospital, Shanghai Jiao Tong University School of Medicine, Shanghai, China; 4 Department of Critical Care Medicine, Shandong Provincial Hospital Affiliated to Shandong First Medical University, Jinan, Shandong China; 5 Department of Critical Care Medicine, The 960th Hospital of the PLA Joint Logistics Support Force, Jinan, Shandong China; 6 Department of Infectious Disease, Provincial Hospital Affiliated to Shandong First Medical University, Jinan, China; 7 Tuberculosis and Respiratory Department, Wuhan Infectious Diseases Hospital, Wuhan, China; 8 Department of Intensive Care Unit, Shandong Provincial Chest Hospital, Jinan, China; 9 Jinan Infectious Diseases Hospital, Shandong University, Jinan, China; 10 General Intensive Care Unit, Raymond Poincaré Hospital (APHP), Laboratory of Inflammation and Infection U1173, FHU SEPSIS, RHU RECORDS, University Paris Saclay-Campus UVSQ, 104 Bd Raymond Poincaré, 92380 Garches, France; 11 Department of Radiology, Jinan People’s Hospital Affiliated to Shandong First Medical University, Jinan, 250021 China

**Keywords:** COVID-19, HBV, Co-infection, Corticosteroid treatment

## Abstract

**Background:**

The impact of corticosteroids on patients with severe coronavirus disease 2019 (COVID-19)/chronic hepatitis B virus (HBV) co-infection is currently unknown. We aimed to investigate the association of corticosteroids on these patients.

**Methods:**

This retrospective multicenter study screened 5447 confirmed COVID-19 patients hospitalized between Jan 1, 2020 to Apr 18, 2020 in seven centers in China, where the prevalence of chronic HBV infection is moderate to high. Severe patients who had chronic HBV and acute SARS-cov-2 infection were potentially eligible. The diagnosis of chronic HBV infection was based on positive testing for hepatitis B surface antigen (HBsAg) or HBV DNA during hospitalization and a medical history of chronic HBV infection. Severe patients (meeting one of following criteria: respiratory rate > 30 breaths/min; severe respiratory distress; or SpO_2_ ≤ 93% on room air; or oxygen index < 300 mmHg) with COVID-19/HBV co-infection were identified. The bias of confounding variables on corticosteroids effects was minimized using multivariable logistic regression model and inverse probability of treatment weighting (IPTW) based on propensity score.

**Results:**

The prevalence of HBV co-infection in COVID-19 patients was 4.1%. There were 105 patients with severe COVID-19/HBV co-infections (median age 62 years, 57.1% male). Fifty-five patients received corticosteroid treatment and 50 patients did not. In the multivariable analysis, corticosteroid therapy (OR, 6.32, 95% CI 1.17–34.24, *P* = 0.033) was identified as an independent risk factor for 28-day mortality. With IPTW analysis, corticosteroid treatment was associated with delayed SARS-CoV-2 viral RNA clearance (OR, 2.95, 95% CI 1.63–5.32, *P* < 0.001), increased risk of 28-day and in-hospital mortality (OR, 4.90, 95% CI 1.68–14.28, *P* = 0.004; OR, 5.64, 95% CI 1.95–16.30, *P* = 0.001, respectively), and acute liver injury (OR, 4.50, 95% CI 2.57–7.85, *P* < 0.001). Methylprednisolone dose per day and cumulative dose in non-survivors were significantly higher than in survivors.

**Conclusions:**

In patients with severe COVID-19/HBV co-infection, corticosteroid treatment may be associated with increased risk of 28-day and in-hospital mortality.

**Supplementary Information:**

The online version contains supplementary material available at 10.1186/s12879-022-07882-6.

## Introduction

The pandemic of COVID-19 induced by severe acute respiratory syndrome coronavirus 2 (SARS-CoV-2) is placing a sustained burden to health care, economic and social systems worldwide [1]. By 2016, there were approximately 292 million people with chronic hepatitis B (CHB) in the world, resulting in severe liver disease [2]. China has a moderate to high incidence of chronic HBV infection, and the prevalence rate of surface antigen in the population is around 4.51–9.51% [3]. COVID-19 may be complicated with acute liver injury. There are still insufficient data on COVID-19/HBV co-infection [4]. Whether the pre-existing chronic HBV infection may aggravate the clinical course of COVID-19 and vice versa is largely unknown [4].

Chronic infection with HBV may result from abnormal host immune responses [2, 3]. Patients with pre-existing HBV infection might be more susceptible to SARS-CoV-2 infection because of an immunocompromised status [5, 6]. In addition, in the presence of co-infection with HBV, immune responses to SARS-CoV-2 may substantially differ from the one observed in immunocompetent patients. Therefore, co-infection with HBV and SARS-CoV-2 might synergistically confer to immune dysfunction and subsequent quite differential immune status during the disease process.

Adults with severe COVID-19 typically present with dysregulated innate and adaptive immune responses resulting in multisystem inflammatory syndrome. Severe COVID-19 patients are characterized by an excessive production of inflammatory cytokines/mediators (IL-6, IL-10, and ferritin) [7]. Patients included in this study were hospitalized at the very beginning of the COVID-19 pandemic, and the effects of corticosteroids treatment were not clear at that time. Whether a patient received corticosteroids or not were largely depend on physicians’ decision. Current WHO guidelines recommend the use of corticosteroids in COVID 19 patients who require oxygen supplementation [8]. However, it remains unclear if the benefit to risk ratio of corticosteroids remains favorable across all subgroups of patients [9, 10]. Thus far, there is little information on efficacy and safety of corticosteroids in the subgroup of patients with severe COVID-19 and HBV co-infection.

We performed a multicenter retrospective study to investigate the effects of treatment with corticosteroids on clinical outcomes in severe COVID-19 patients with chronic HBV co-infection.

## Patients and methods

### Study design and participants

This is a retrospective study enrolling patients hospitalized between Jan 1, 2020 to Apr 18, 2020, in 7 medical centers (including Wuhan Huoshenshan Hospital, Wuhan Infectious Diseases Hospital, Wuhan Ninth People’s Hospital, Wuhan Fourth People’s hospital, Hubei Huanggang Central Hospital, Shandong Provincial Chest Hospital and Shandong Infectious Diseases Hospital). The ethics committee of all participating institutions approved the study protocol.

Inclusion criteria were patients who fulfilled confirmed diagnosis of severe COVID-19 and chronic HBV at admission. The diagnosis of chronic HBV infection was based on a medical history of chronic HBV infection and positive testing for hepatitis B surface antigen (HBsAg) or HBV DNA [11]. Patients with COVID-19 were considered to have severe illness if they met at least one of following criteria [12]: respiratory rate > 30 breaths/min; severe respiratory distress; or SpO_2_ ≤ 93% on room air, or oxygen index < 300 mmHg.

### Data collection and study outcomes

Data extraction was performed by a trained team of physicians using a standardized form to collect data from electronic medical records on demographic characteristics, medical history, underlying medical conditions, symptoms and signs from disease onset to hospital admission, complications and outcomes, laboratory tests and treatments. All recorded data were double-checked by trained physicians and a third researcher adjudicated any discrepancies.

The primary outcomes were all cause mortality at 28-day from hospital admission and hospital discharge. The secondary outcomes were development of acute respiratory distress syndrome (ARDS), sepsis shock, acute liver injury, acute kidney injury (AKI), acute cardiac injury, the need for invasive mechanical ventilation, for continuous renal replacement therapy (CRRT), and the time from symptoms onset to SARS–CoV-2 RNA clearance in respiratory secretions.

### Statistical analysis

The Kolmogorov–Smirnov test or Shapiro–Wilk test was used to test the normality for continuous variables. Continuous variables with normal distribution were expressed as mean ± SD and compared using unpaired, 2-tailed Student’s t test. Continuous variables with skewed distribution were presented as median (interquartile range) and compared with Mann–Whitney U test. Categorical variables were summarized as numbers (percentages) and compared by Pearson Chi-square test or Fisher’s exact test. Kaplan–Meier estimator was constructed to estimate the survival curves over 28-day period and log-rank test was used to compare the survival probability between corticosteroid treatment group and non-corticosteroid treatment group. To explore the risk factors associated with 28-day mortality, univariate analysis and multivariable logistic regression model were constructed to estimate the OR and 95% confidence interval (95% CI). The variables in the multivariable logistic regression model were as follows: Lymphocyte count, Hs-CRP, age, gender, ALT, comorbidity, albumin and d-dimer on hospital admission and corticosteroid treatment initiation respectively, all of which were selected based on existing literatures [13, 14], and significance of the *P* value in the univariate analysis according to the data in this study.

To confirm the association of corticosteroid therapy on mortality, we performed three analytic strategies to minimize the bias introduced by confounding variables. First, we performed IPTW analysis based on propensity score to estimate causal treatment effects. To this purpose, propensity score for each patient was calculated by logistic regression model that included the same variables that had been used in the priori logistic regression model. In both unweighted and pseudo-population cohorts, the standardized mean difference (SMD) was computed. An SMD of > 10% suggested an imbalance between groups. Second, we constructed an extended Cox regression model which incorporated the same above-mentioned confounding variables and corticosteroid therapy as a time-varying exposure variable, as previously described [15, 16]. Third, we constructed a multivariable logistic regression model which incorporated corticosteroid therapy as a categorical (yes/no) variable and the same confounding variables with the values at the time of corticosteroid therapy initiation (not the values at the baseline) to avoid the issue of “indication bias”. The time of variable drawn from patients without corticosteroids was according to the median initiation time of corticosteroid treatment.

In addition, the association of corticosteroid therapy on 28-day mortality was analyzed in six predefined subgroups: male vs. female; age ≥ 65 years vs. age < 65 years; lymphocyte < 0.8 × 10^9^/L vs ≥ 0.8 × 10^9^/L; d-dimer < 1 µg/mL vs ≥ 1 µg/mL; albumin < 30 g/L vs ≥ 30 g/L, Hs-CRP < 5 mg/L vs ≥ 5 mg/L. The cut-off value for continuous variables in each subgroup was determined according to previous clinical constraints [13, 14, 17]. In subgroups, OR with 95% CI were estimated by logistic regression analysis. Numerical missing data was imputed by median and categorical data was imputed by the category with the most frequency. A two-tailed *P* value of 0.05 or less was considered statistically significant. Statistical analyses were done using SPSS software, version 22.0 (SPSS Inc. Chicago, Illinois, United States), SAS9.4, and R 3.6.2 (R Foundation for Statistical Computing).

## Results

### Demographic and clinical characteristics of patients with COVID-19/HBV co-infection

**Fig. 1:**
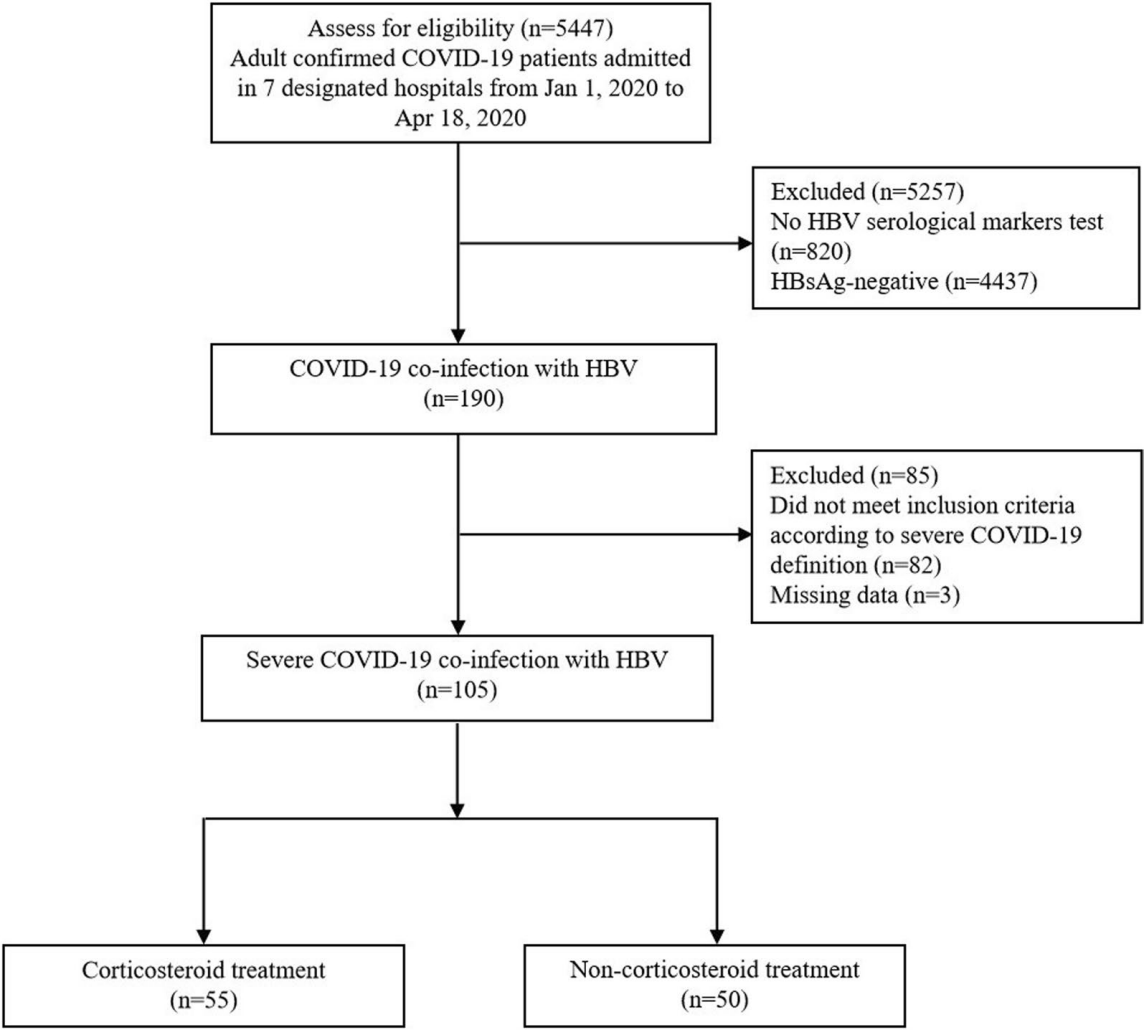
Flow diagram of the progress of the trial. COVID-19: coronavirus disease 2019; HBV: hepatitis B virus

A total of 5447 adult patients with confirmed COVID-19 were screened, we excluded 820 without results of HBV serological marker test. Among 4627 remainders, 190 patients were HBsAg-positive. The prevalence of HBV co-infection in hospitalized COVID-19 patients was 4.1% (Fig. 1).

Of the 190 cases with COVID-19 and HBV co-infection, 105 fulfilled the definition of severe COVID-19. The incidence of severe cases in COVID-19 and HBV co-infection patients was 55.3%. Among 105 patients with COVID-19 and HBV co-infection, 55 received corticosteroid treatment (n = 55) and 50 did not (n = 50) (Fig. 1).

Baseline characteristics were comparable in the two groups. The median age was 62.0 years (IQR 54.0–71.0). Sixty (57.1%) patients were male. Fifteen patients had pre-existing cirrhosis. A majority of patients were tested negative for HBeAg (95.2%) (100/105). The incidence of HBeAb positive was 17.1% (18/105) (Table 1).

**Table 1:** Baseline characteristics of patients with severe COVID-19 and HBV co-infection

Characteristics	All patients N = 105	Survivors N = 87	Non-survivors N = 18	*P* value
Median age (IQR), year	62 (54,71)	62 (53, 70)	70 (58,75)	0.024
Gender, n (%)				
Female	45 (42.9)	39 (44.8)	6 (33.3)	0.370
Male	60 (57.1)	48 (55.2)	12 (66.7)	
Comorbidity				
Diabetes, n (%)	16 (15.2)	12 (13.8)	4 (22.2)	0.585
Hypertension, n (%)	38 (36.2)	29 (33.3)	9 (50.0)	0.180
Coronary heart disease, n (%)	11 (10.5)	8 (9.2)	3 (16.7)	0.603
COPD, n (%)	5 (4.8)	2 (2.3)	3 (16.7)	0.034
Cancer, n (%)	9 (8.6)	6 (6.9)	3 (16.7)	0.376
Cirrhosis, n (%)	15 (14.3)	11 (12.6)	4 (22.2)	0.492
HBV infection, n (%)				
HBeAg positive, n (%)	5 (4.8)	3 (3.4)	2 (11.1)	0.206
HBeAb positive, n (%)	18 (17.1)	16 (18.4)	2 (11.1)	0.659
HBcAb positive, n (%)	105 (100)	87 (100)	18 (100)	> 0.999
Anti-HBV treatment history, n (%)	8 (7.6)	5 (5.7)	3 (16.7)	0.271
Time from symptom onset to admission, median (IQR), days	14 (8, 20)	14 (8, 21)	10 (7, 16)	0.247
Drugs treatment during hospitalization				
Corticosteroid treatment, n (%)	55 (52.4)	39 (44.8)	16 (88.9)	0.001
IFN-α, n (%)	16 (15.2)	13 (14.9)	3 (16.7)	0.853
Thymosin treatment, n (%)	29 (27.6)	22 (25.3)	7 (38.9)	0.240
Anti-HBV treatment, n (%)	9 (8.6)	8 (9.2)	1 (5.6)	0.968
Gamma globulin treatment, n (%)	36 (34.3)	25 (28.7)	11 (61.1)	0.008
Anticoagulant therapy, n (%)	21 (20.0)	14 (16.1)	7 (38.9)	0.028

COVID-19: coronavirus disease 2019, HBV: hepatitis B virus, IQR: interquartile range, IFN-α: interferon-α

*P* values indicate differences between survivors and non-survivors. *P* < 0.05 was considered statistically significant

A higher proportion of corticosteroids treated patients received therapeutic anticoagulants (29.1% vs 10.0%, *P* = 0.015) (Additional file 1: Table S2).

The median initiation time of corticosteroid treatment was 2 (1, 5) days after admission.

### Laboratory findings at baseline and at the time of corticosteroid therapy initiation

The baseline laboratory data and characteristics were displayed in Additional file 1: Tables S1–S3. There was no significant difference between corticosteroids treated and corticosteroids free patients for leukocytes and platelets counts, and plasma levels of d-dimer, ALT, AST, ALP, bilirubin, pre-albumin, albumin, total cholesterol, triglyceride, high density lipoprotein, high-sensitivity troponin, IL-6. In addition, SOFA and APACHE II scores at baseline were not statistically different between the two groups (Additional file 1: Table S3). Laboratory findings at the time of corticosteroid therapy initiation were displayed in Additional file 1: Table S4. Likewise, there was no significant difference between corticosteroids treated and corticosteroids free patients for above mentioned laboratory parameters.

### Association of corticosteroid treatment in critically ill patient with COVID-19 and HBV co-infection

#### Primary outcomes

**Fig. 2:**
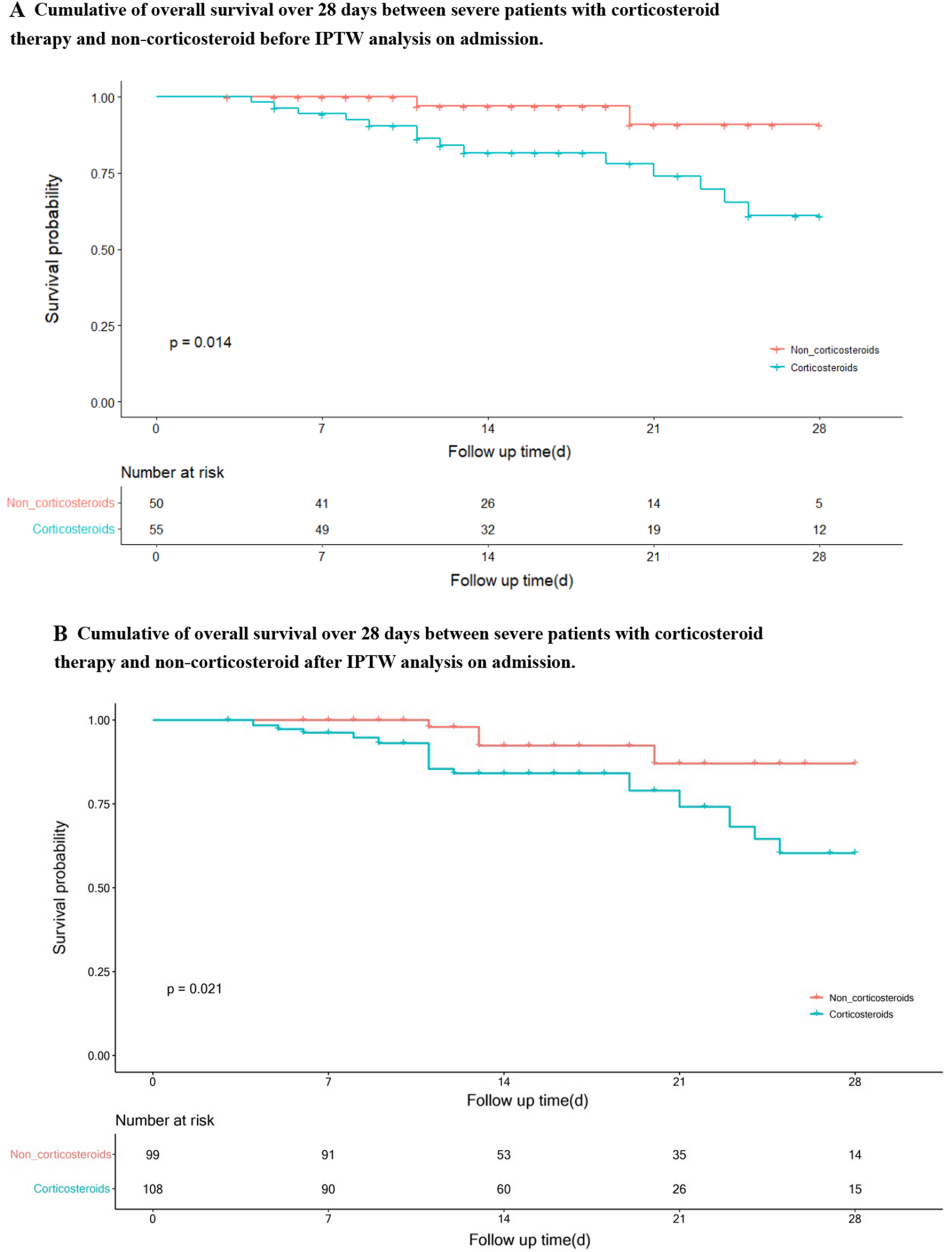
**A** Cumulative of overall survival over 28 days between severe patients with corticosteroid therapy and non-corticosteroid before IPTW analysis on admission. **B** Cumulative of overall survival over 28 days between severe patients with corticosteroid therapy and non-corticosteroid after IPTW analysis on admission

The survival rate was significantly lower in the corticosteroids group (41/55, 74.5% vs 48/50, 96.0%, *P* = 0.014, log-rank test) (Fig. 2A, Table 2). By univariate analysis, age, lymphopenia, d-dimer greater than 1 μg/mL, albumin less than 30 g/L on admission and corticosteroid treatment were associated with 28-day mortality (Table 3). By multivariable logistic regression analysis, corticosteroid treatment (OR, 6.32, 95% CI 1.17–34.24, *P* = 0.033) were independent risk factors for 28-day mortality (Table 3).

**Table 2:** Clinical course and outcomes of patients with severe COVID-19 and HBV co-infection according to receiving corticosteroid therapy or not

Characteristics	All patients N = 105	Corticosteroid N = 55	Non-corticosteroid N = 50	*P* value
ARDS (n, %)	105 (100)	55 (100)	50 (100)	> 0.999
Sepsis shock (n, %)	28 (26.7)	11(20.0)	17 (34.0)	0.105
Acute liver injury after corticosteroid initiation (n, %)	52 (49.5)	33 (60.0)	19 (38.0)	0.024
Acute kidney injury (n, %)	16 (15.2)	9 (16.4)	7 (14.0)	0.736
Acute cardiac injury (n, %)	18 (17.1)	10 (18.2)	8 (16.0)	0.767
Invasive mechanical ventilation, n (%)	19 (18.1)	9 (16.4)	10 (20.0)	0.629
Non-invasive mechanical ventilation, n (%)	14 (13.3)	8 (14.5)	6 (12.0)	0.702
High-flow nasal oxygen, n (%)	16 (15.2)	9 (16.4)	7 (14.0)	0.736
Nosocomial infection, n (%)	14 (13.3)	6 (10.9)	8 (16.0)	0.443
CRRT (n, %)	4 (3.8)	2 (3.6)	2 (4.0)	> 0.999
ECMO (n, %)	1 (1.0)	1 (1.8)	0 (0)	> 0.999
ICU admission (n, %)	44 (41.9)	25 (45.5)	19 (38.0)	0.439
Discharged from hospital within 28 days, n (%)	89 (84.8)	44 (80.0)	45 (90.0)	0.154
28-day mortality (n, %)	16 (15.2)	14 (25.5)	2 (4.0)	0.005
In-hospital mortality (n, %)	18 (17.1)	16 (29.1)	2 (4.0)	0.002
Time from symptom onset to discharge or death, median (IQR), days	25 (20, 30)	24 (19,33)	26 (22, 30)	0.355
SARS-CoV-2 RNA positive more than 20 days (n, %)	41 (39.0)	32 (58.2)	9 (18.0)	< 0.001
Time from symptom onset to SARS-Cov-2 RNA negative, median (IQR), days	22 (16, 24)	24 (19, 27)	17 (12, 22)	0.026

COVID-19: coronavirus disease 2019; HBV: hepatitis B virus; ARDS: acute respiratory distress syndrome; IQR: inter quartile range; CRRT: continuous renal replacement therapy; ECMO: extracorporeal membrane oxygenation

*P* values indicate differences between corticosteroid and non-corticosteroid. *P* < 0.05 was considered statistically significant

**Table 3:** Logistic regression model evaluating risk factors associated with 28-day mortality in severe patients with COVID-19 and HBV co-infection (on admission)

Variables	28-day mortality			In-hospital mortality			Acute liver injury			Acute kidney injury			Acute cardiac injury		
	OR	95% CI	*P* value	OR	95% CI	*P* value	OR	95% CI	*P* value	OR	95% CI	*P* value	OR	95% CI	*P* value
Age	1.067	1.003–1.135	0.041	1.078	1.016–1.143	0.013									
Gender (Male vs Female)							2.256	1.015–5.013	0.046						
Comorbidity													4.737	1.264–17.745	0.021
Lymphocyte count < 0.8 × 10^9^/L	5.933	1.440–24.450	0.014												
d-dimer > 1 μg/mL	5.780	1.478–22.610	0.012							2.232	1.042–3.950	0.043			
Albumin < 30 g/L				5.410	1.444–20.269	0.012									
Corticosteroid treatment	6.318	1.166–34.239	0.033	11.743	2.299–59.992	0.003									

Comorbidity: Other underlying diseases excluding HBV infection; ALT: alanine amino transferase; Hs-CRP: High sensitive c reaction protein; OR: odds ratio; CI: confidence intervals

**Fig. 3:**
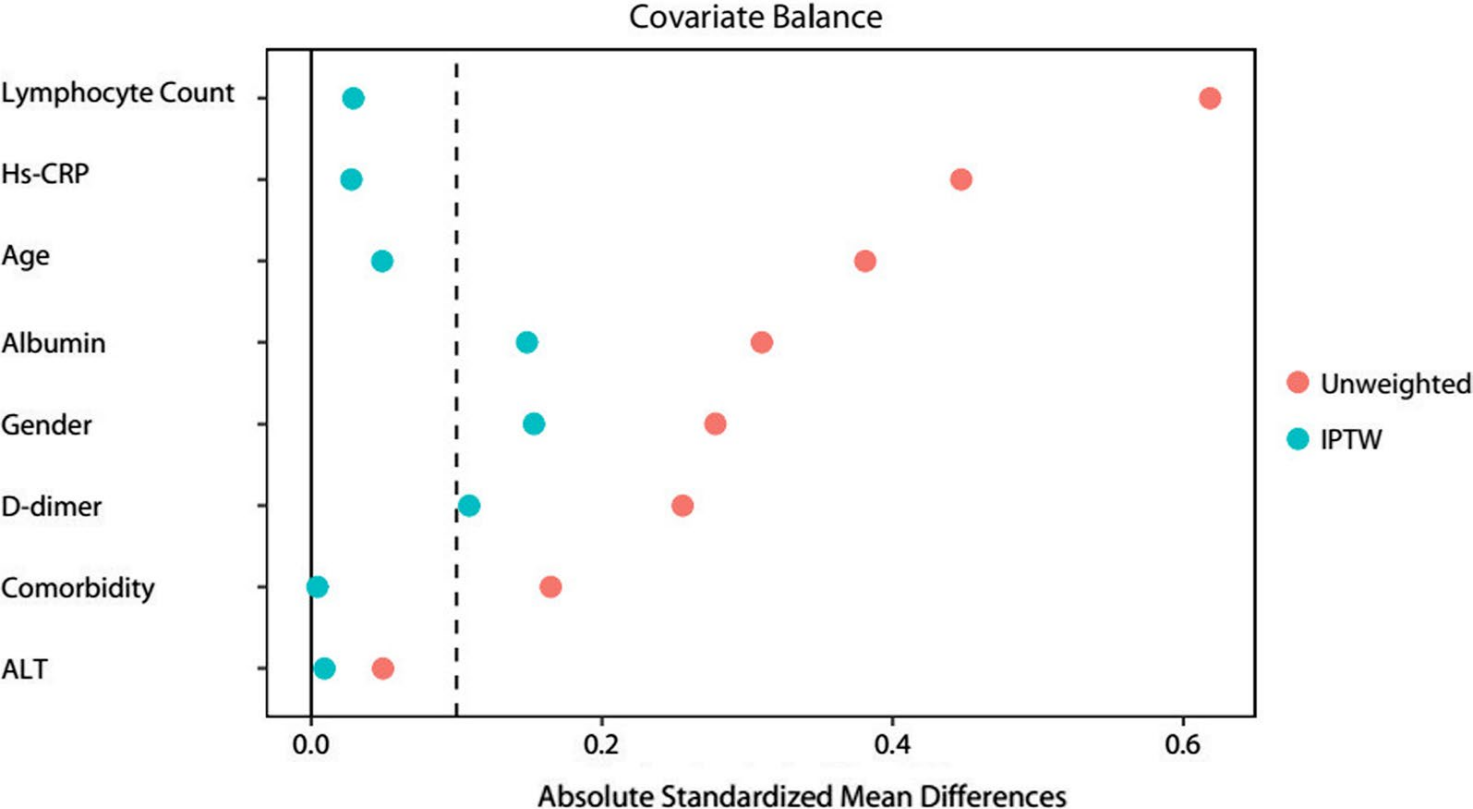
Subgroup analysis of 28-day mortality according to corticosteroid therapy in patients with severe COVID-19 and HBV co-infection. COVID-19: coronavirus disease 2019; HBV: hepatitis B virus; Hs-CRP: high sensitive c reaction protein; RR: risk ratio; CI: confidence interval

**Fig. 4:**
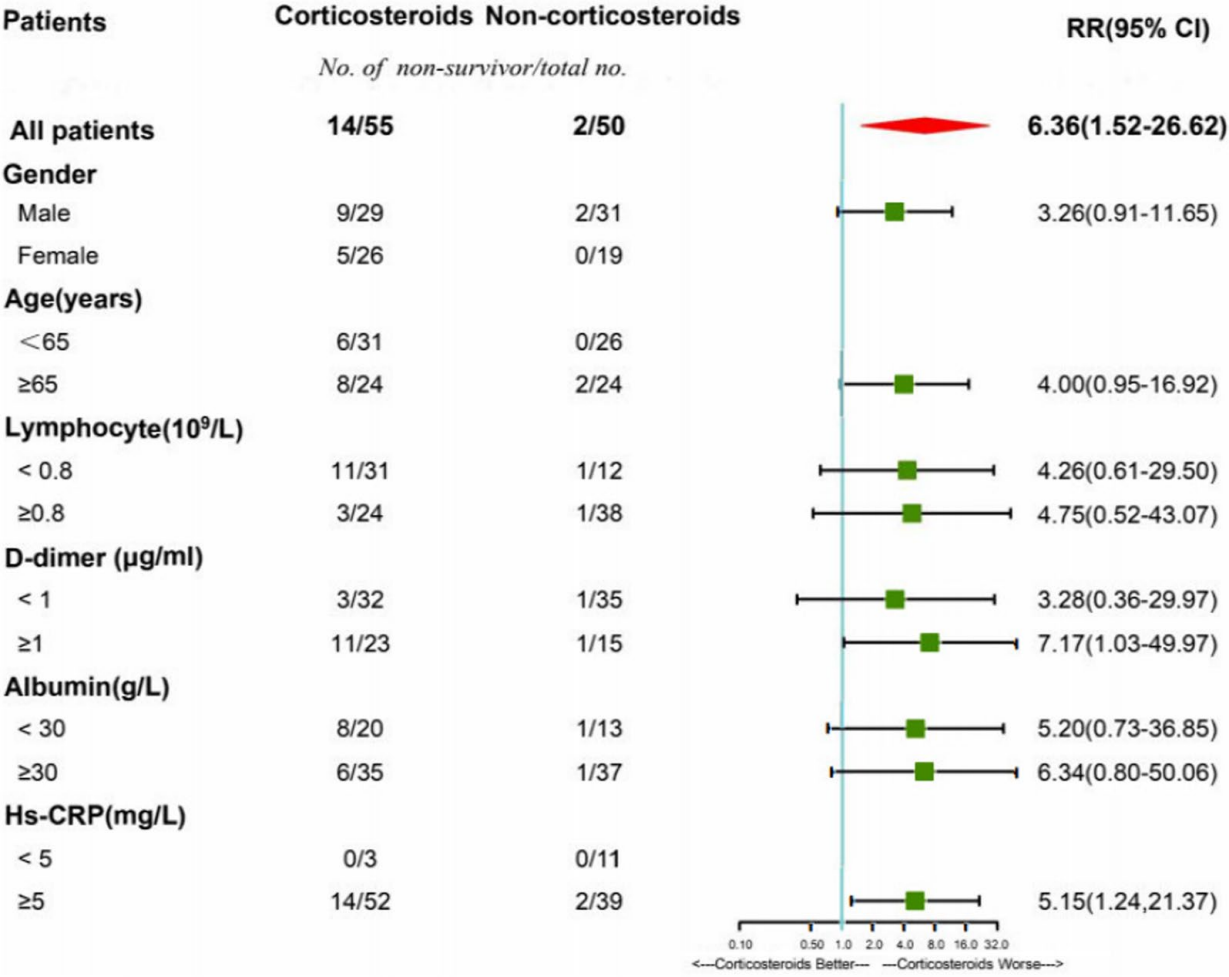
Summaries of the balance of variables before and after propensity score matching. Standardized Mean Difference before and after IPTW using the variables with the values at baseline (on admission). Hs-CRP: High sensitive c reaction protein; ALT: Alanine amino transferase

Using different analytic strategies for adjustment yielded highly consistent results, including the IPTW analysis (OR, 4.90, 95% CI 1.68–14.28, *P* = 0.004) (Figs. 2B, 3 and Table 4); the extended Cox regression model which treated corticosteroid therapy as a time-varying exposure variable (HR 4.68, 95% CI 1.44–15.25, *P* < 0.001); the multivariable logistic regression model which incorporated variables with the values at the time of corticosteroid therapy initiation (OR 7.13, 95% CI 1.35–37.56, *P* = 0.021) (Additional file 1: Figures S1, S2 and Tables S5, S6). In the subgroup analysis, the associations between corticosteroid therapy and mortality were not significantly changed with varying subpopulations based on gender, age, lymphocyte, d-dimer, albumin and Hs-CRP (Fig. 4).

**Table 4:** The comparison of primary and secondary outcomes of patients with severe COVID-19 and HBV co-infection according to corticosteroids and non-corticosteroids treatment after IPTW analysis (on admission)

Outcome	OR	95% CI	*P* value
Primary outcomes			
28-day mortality	4.901	1.682–14.283	0.004
In-hospital mortality	5.637	1.949–16.299	0.001
Secondary outcomes			
Acute liver injury	4.495	2.574–7.852	< 0.001
Acute kidney injury	1.031	0.466–2.278	0.940
Acute cardiac injury	1.163	0.554–2.439	0.690
Invasive mechanical ventilation	0.481	0.226–1.023	0.057
CRRT	0.230	0.032–1.653	0.144
SARS-CoV-2 RNA positive more than 20 days	2.945	1.631–5.317	< 0.001
Sepsis shock	0.384	0.202–0.730	0.004
ARDS			> 0.999

COVID-19: coronavirus disease 2019; HBV: hepatitis B virus; CRRT: continuous renal replacement therapy; SARS-CoV-2: severe acute respiratory syndrome coronavirus 2; ARDS: acute respiratory distress syndrome; OR: odds ratio; CI: confidence intervals

*P* values indicate differences between corticosteroid and non-corticosteroid. *P* < 0.05 was considered statistically significant

#### Secondary outcomes

More patients had SARS-CoV-2 RNA positive result in upper respiratory tract more than 20 days after symptoms onset in patients treated with versus without corticosteroids (58.2% vs 18.0%, *P* < 0.001) (Table 2).

The IQR time from symptoms onset to SARS-CoV-2 RNA clearance was longer in corticosteroids treated versus corticosteroids-free patients (IQR: 24 days vs 17 days, *P* = 0.026) (Table 2).

There was no significant difference between corticosteroids treated and corticosteroids free patients for the incidence of septic shock, AKI and acute cardiac injury. However, corticosteroids treatment was associated with increased risk of acute liver injury (60.0% vs 38.0%, *P* = 0.024) (Table 2), and this result was confirmed by using other analytic strategies, including IPTW analysis (OR 4.50, 95% CI 2.57–7.85, P < 0.001) (Table 4) and the multivariable logistic regression model which incorporated variables with the values at the time of corticosteroid therapy initiation (OR, 1.85, 95% CI 1.07–3.20, *P* = 0.029) (Additional file 1: Table S6).

#### Laboratory parameters

**Fig. 5:**
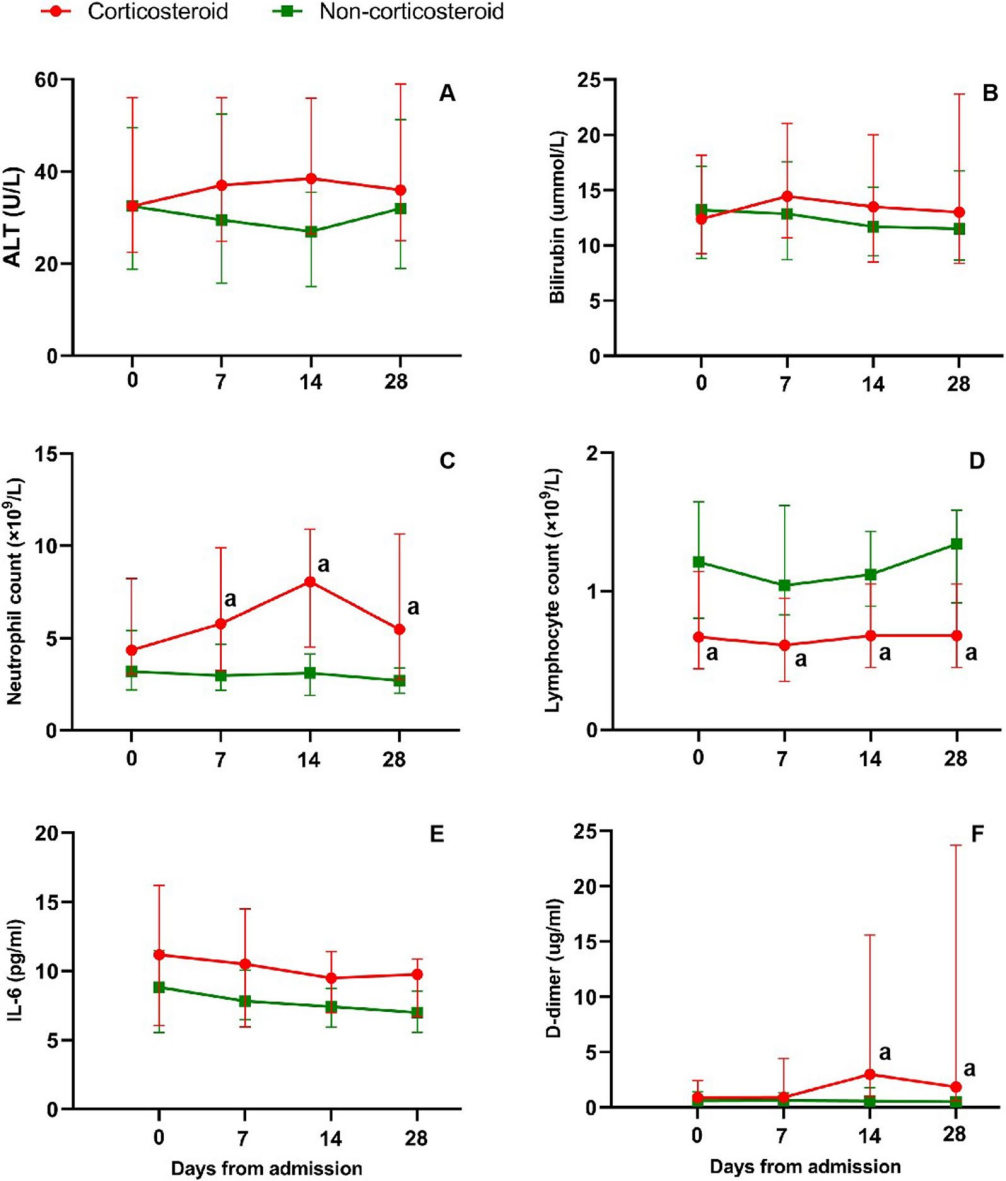
Dynamic profile of laboratory markers in patients with severe COVID-19 and HBV co-infection from illness onset (non-corticosteroid treatment vs corticosteroid treatment). This figure shows dynamic profile in ALT (**A**), Bilirubin (**B**), Neutrophil count (**C**), Lymphocyte count (**D**), IL-6 (**E**), and d-dimer (**F**) from the onset of the disease to 28 days. The bars represent interquartile range. ^a^*P *< 0.05 indicate differences between non-corticosteroid treatment vs corticosteroid treatment. COVID-19: coronavirus disease 2019; HBV: hepatitis B virus; ALT: alanine amino transferase; IL-6: interleukin-6

As compared to corticosteroids-free patients, corticosteroids treated patients had significantly increased neutrophils counts and d-dimer levels (time points were 14 days, 28 days after admission respectively) (all *P* < 0.05) (Fig. 5C, F). Corticosteroids treatment decreased lymphocyte counts (*P* < 0.05) (Fig. 5D). Serum levels for ALT, bilirubin and IL-6 were not statistically different during corticosteroid treatment (time points were 7 days, 14 days, and 28 days after admission respectively) (all *P* > 0.05) (Fig. 5A, B, E).

### Corticosteroid therapy among patients with severe COVID-19 and HBV co-infection

Most patients (48/55, 87.3%) received corticosteroid therapy more than 7 days after symptoms onset, including 15 non-survivors (Table 5). All patients in corticosteroid treatment group received methylprednisolone. In subgroup analysis, methylprednisolone average dose was significantly higher in non-survivors (83 mg/day) than in survivors (40 mg/day) (Table 5).

**Table 5:** Corticosteroid therapy among patients with severe COVID-19 and HBV co-infection

Characteristics	All patients N = 55	Survivor N = 39	Non-survivor N = 16	*P* value
Time from symptom onset to corticosteroid initiation, median (IQR), days	15 (10, 19)	15 (10, 25)	14 (9, 17)	0.500
< 7 days, n (%)	7 (12.7)	6 (15.4)	1 (6.3)	0.633
** ≥ **7 days, n (%)	48 (87.3)	33 (84.6)	15 (93.8)	
Time from ARDS onset to corticosteroid initiation, median (IQR), days	0 (− 1, 1)	0 (− 1, 0)	0 (− 2, 5)	0.324
< 3 days, n (%)	28 (50.9)	19 (48.7)	9 (56.3)	0.612
** ≥ **3 days, n (%)	27 (49.1)	20 (51.3)	7 (43.8)	
Duration of corticosteroid, median (IQR), days	5 (1, 7)	5 (1, 7)	6 (2, 10)	0.396
< 7 days, n (%)	42 (76.4)	32 (82.1)	10 (62.5)	0.121
** ≥ **7 days, n (%)	13 (23.6)	7 (17.9)	6 (37.5)	
Cumulative dosage during the whole course of treatment	248(105,460)	200 (102, 280)	430 (236, 1094)	0.014
< 400 mg, n (%)	41 (74.5)	33 (84.6)	8 (50.0)	0.007
** ≥ **400 mg, n (%)	14 (25.5)	6 (15.4)	8 (50.0)	
Dose per day (mg)	40 (40, 80)	40 (40,46)	83 (50, 106)	0.113
< 80	42 (76.4)	34 (87.2)	8 (50.0)	0.003
** ≥ **80	13 (23.6)	5 (12.8)	8 (50.0)	
Time from symptom onset to discharge or death, median (IQR), days	24 (19, 33)	24 (19, 36)	22 (18,27)	0.133
Time from corticosteroid initiation to discharge or death, median (IQR), days	14 (6, 21)	14 (7, 23)	11 (4, 19)	0.453

COVID-19: coronavirus disease 2019; HBV: hepatitis B virus; ARDS: acute respiratory distress syndrome; IQR: interquartile range

*P* values indicate differences between survivors and non-survivors. *P* < 0.05 was considered statistically significant

### General clinical characteristics of non-survivors with COVID-19/HBV co-infection

Causes of death of 18 non-survivors with COVID-19/HBV co-infection were listed in Additional file 1: Table S5. Patients from No. 1 to 16 received corticosteroid treatment and patient No. 17, 18 did not. Twelve patients were of male gender. Age ranged from 46 to 83 years. 15 patients with corticosteroid treatment had very high d-dimer levels. In these patients, main causes of death were severe ARDS and multiple organs failure, including liver, cardiac and kidney dysfunction.

## Discussion

To the best of our knowledge, this is the first report on the clinical impact of corticosteroid treatment on patients with severe COVID-19/HBV co-infection. We retrospectively reviewed and found that corticosteroid treatment was associated with higher mortality in patients with severe COVID-19 and HBV co-infection. Furthermore, doses of 83 mg/day or more of methylprednisolone, and initiation after 7 days from first symptoms may be associated with increased mortality. Survivors in corticosteroid group received corticosteroid therapy with lower cumulative dose (< 400 mg methylprednisolone) and daily dose (< 80 mg methylprednisolone). Our study showed that corticosteroid treatment was associated with higher d-dimer level and neutrophils count. The proportion of patients with corticosteroid therapy receiving therapeutic anticoagulants was higher than in corticosteroids-free patients. These results may contribute in the identification of subgroups of patients with COVID 19 who may not receive corticosteroids. A recent report found that the prevalence rate of HBV in the general population was 7–11%, while that of COVID-19 patients was only 0–1.3%. By contrast, in our cohort of COVID-19 patients, the prevalence of HBV was 4.1%. Corticosteroids were more likely to be given to in patients with severe COVID-19. Patients in this cohort study were hospitalized at the very beginning of the COVID-19 pandemic and the evidences on corticosteroid therapy were limited. Physician made decision to implement corticosteroid therapy or not among severe patients based on their experiences. The current management of patients with severe COVID-19 has substantially changed. The UK-based Randomized Evaluation of COVID-19 Therapy (RECOVERY) trial reported that dexamethasone reduced mortality by one-third (29.3% vs 41.4% for usual care) in severe COVID-19 patients who required respiratory support [18]. One meta-analysis of clinical trials found that compared with usual care or placebo, systemic corticosteroid treatment was associated with lower 28-day mortality [9]. To date, there is no definite recommendation on whether corticosteroids should be used or not in patients with severe COVID-19 and HBV co-infection. Our findings suggested that in these patients, corticosteroids may be associated with increased short-term mortality.

One explanation for worse outcomes with corticosteroids in patients with COVID-19 and HBV co-infection may be a combination of HBV and SARS-CoV-2 mediated effects and immune response. Chronic HBV infection is characterized by dysfunction of innate and adaptive immune response, particularly a deficiency in virus-specific CD8+ T cells [15]. The function of B cells producing antibodies in HBV infection is also impaired [16]. The decrease in immune cells, especially lymphocytes, CD4+ T cells and CD8+ T cells is a marker of poor prognosis in COVID-19 patients [19]. This is consistent with our results of decreased lymphocyte count in non-survivors. Therefore, immune deficiency caused by chronic HBV infection may play a role in the progression of COVID-19 disease. The immune-suppressing effects of corticosteroid therapy, which are mediated mainly by T-cell responses [20], may exacerbate the immune dysfunction in patients with COVID-19 and HBV co-infection. It was found that the immune deficiency may affect the immune response to SARS-COV-2 resulting in delayed viral clearance [5, 21]. In our study, 58.2% corticosteroid treated patients still had detectable SARS-CoV-2 RNA in upper respiratory tract after 20 days from the onset of symptoms, which was significantly more than in corticosteroids free patients (18.0%). In COVID-19 with HCV and HIV, it was also found that immune-deficiency would alter host response to SARS-COV-227. Therefore, immune deficiency may relate to clinical course of COVID-19 and HBV co-infection after receiving corticosteroid therapy. Similarly, observational studies in patients with SARS and MERS suggested that corticosteroid therapy was associated with delayed viral clearance from blood and respiratory tract and increased risk of secondary infection [22, 23]. Our results were consistent with previous studies and further supported a role of corticosteroids in prolonging SARS-CoV-2 replication in patients with COVID-19 and HBV co-infection.

As an immune suppressive drug, corticosteroid therapy is a risk factor of HBV reactivation in chronic HBV infection patients [24]. Hepatitis B reactivation is the reappearance or rise of HBV DNA in the serum of patients with past or chronic HBV infection [24]. It may result in fulminant hepatitis and may cause death. Recently, a large size observational study showed that patients who had a history of HBV infection and received systemic corticosteroids with high peak daily doses (> 40 mg prednisolone equivalent) had a higher risk of hepatitis flare, although the mortality was not significantly increased. It should be cautious to systematically use corticosteroids in patients with HBV infection [25]. In our study, corticosteroids were associated with increased incidence of acute live injury, suggesting altered liver function. Liu et al. found that COVID-19 patients co-infected with chronic HBV could have a risk of hepatitis B reactivation, especially in patients with corticosteroid therapy [5]. A majority of patients in this cohort study were HBeAg-negative CHB. HBV DNA was detected in nearly one-third patients and did not find positive results. Most patients in this cohort study might be HBV portage and active chronic infection was infrequent. Since most patients in this cohort did not undergo multiple HBV DNA tests, further studies are needed to determine whether these multiple organ function injuries are related to HBV reactivation. In addition, patients with HBV related cirrhosis have very poor immune function and liver function [6]. Pre-existing cirrhosis increased the risk of poor outcome related to COVID-19. Only 15 patients had cirrhosis in our study and 11 survived. Therefore, the effects of different stages of HBV infection on the prognosis of COVID-19 patients need to be further studied.

Furthermore, this study provided preliminary evidence for the association of corticosteroids on laboratory findings in severe COVID-19/HBV co-infection patients. We found that corticosteroid treatment was associated with increased d-dimer levels. SARS-CoV-2 infection induced coagulopathy and secondary hyper-fibrinolysis [26, 27]. Autopsy in COVID-19 found systemic microvascular thrombosis in most cases [27, 28]. Higher d-dimer levels on admission could effectively predict in-hospital mortality in COVID-19 patients as well as persistent elevated levels [28]. In our study, most of non-survivors had high d-dimer levels. In patients with severe COVID-19 and chronic HBV co-infection, corticosteroids might increase risk of coagulopathy and thrombosis.

Our study has several limitations. First, the small sample size generated a wide confidence interval may result in imprecision of the effects estimation. Although the results from different models consistently suggested a harm effects of corticosteroid therapy on 28-mortality, future large-scale and multi-center studies are warranted to validate our findings. Second, missing data on HBV-DNA levels prevented us analyzing the association of corticosteroids according to various clinical phases of chronic HBV infections (active infection versus carriage). We did not know the exact rate of HBV infection for all patients included in this study, and 820 patients were excluded owing to the lack of HBV serological testing. Third, we were unable to explore if the liver injury was associated with concurrent drug therapies for COVID-19. Forth, only a small proportion of patients received nucleotide/nucleoside analogue therapy which precluded assessment of the impact of nucleotide/nucleoside analogue therapy on liver function and outcomes. Five, patients in our study arrived at hospital late and the median time of admission was 14 days. Therefore, some patients received corticosteroids therapy in the late stage, which might cause bias impact on results estimation.

## Conclusions

In patients with severe COVID-19 and HBV co-infection, corticosteroid treatment was associated with increased risk for 28-day and in-hospital mortality, high d-dimer level, neutrophil count and acute liver injury, and delayed SARS-CoV-2 viral RNA clearance. There is a risk that corticosteroid treatment and severe disease are interconnected, therefore it should be addressed in a placebo-controlled randomized trial in the future.

### Implications for clinical practice and future research

COVID-19 and HBV co-infection is not infrequent and there is an urgent need to warn about the potential risk associated with corticosteroid treatment in this subgroup of patients. This multicenter study may warn physicians and hepatologists about the potential detrimental effects of corticosteroid therapy on patients with severe COVID-19 and chronic HBV co-infection. The clinical features and the underlying mechanism of different response to corticosteroid therapy in severe COVID-19 patients with chronic HBV co-infection need to further investigations in the context of recommendations to use corticosteroids in the routine management of COVID-19 requiring oxygen supplementation.

## Supplementary Information

**Additional file 1: Table S1. **The laboratory findings of patients with severe COVID-19 and HBV co-infection on admission. Values are median (IQR) unless stated otherwise. **Table S2.** Characteristics of patients with severe COVID-19 and HBV co-infection on admission according to receiving corticosteroid therapy or not. **Table S3.** The laboratory findings of patients with severe COVID-19 and HBV co-infection on admission according to receiving corticosteroid therapy or not. Values are median (IQR) unless stated otherwise. **Table S4.** The laboratory findings of patients with severe COVID-19 and HBV co-infection according to receiving corticosteroid therapy or not on the time of corticosteroid therapy initiation. Values are median (IQR) unless stated otherwise. **Table S5.** Logistic regression modeling evaluating risk factors associated with 28-day mortality in patients with severe COVID-19 and HBV co-infection (on the time of corticosteroid therapy initiation). **Table S6.** The primary and secondary outcomes of patients with severe COVID-19 and HBV co-infection between corticosteroids and non-corticosteroids treatment after IPTW analysis (on time of corticosteroid therapy initiation). **Table S7.** General characteristics and cause of death of severe patients with COVID-19 and HBV co-infection (16 corticosteroids treatment and 2 non-corticosteroids treatment). **Figure S1.** Cumulative of overall survival over 28 days between severe patients with corticosteroid therapy and non-corticosteroid after IPTW analysis on the time of corticosteroid therapy initiation. **Figure S2.** Standardized mean difference before and after IPTW using the variables at the time of corticosteroids initiation.

## Data Availability

The data that support the findings of this study are available from the corresponding author on reasonable request. Participant data without names and identifiers will be made available after approval from the corresponding author. After publication of study findings, the data will be available for others to request. The research team will provide an email address for communication once the data are approved to be shared with others.
