# Supplementary material for: Corticosteroid treatment in severe patients with SARS-CoV-2 and chronic HBV co-infection: a retrospective multicenter study
Source: BMC Infect Dis. 2022 Nov 28;22:891. doi: 10.1186/s12879-022-07882-6 (PMC9702873; doi:10.1186/s12879-022-07882-6)
Supplement: Supplementary file 1 — Additional file 1: Table S1. The laboratory findings of patients with severe COVID-19 and HBV co-infection on admission. Values are median (IQR) unless stated otherwise. Table S2. Characteristics of patients with severe COVID-19 and HBV co-infection on admission according to receiving corticosteroid therapy or not. Table S3. The laboratory findings of patients with severe COVID-19 and HBV co-infection on admission according to receiving corticosteroid therapy or not. Values are median (IQR) unless stated otherwise. Table S4. The laboratory findings of patients with severe COVID-19 and HBV co-infection according to receiving corticosteroid therapy or not on the time of corticosteroid therapy initiation. Values are median (IQR) unless stated otherwise. Table S5. Logistic regression modeling evaluating risk factors associated with 28-day mortality in patients with severe COVID-19 and HBV co-infection (on the time of corticosteroid therapy initiation). Table S6. The primary and secondary outcomes of patients with severe COVID-19 and HBV co-infection between corticosteroids and non-corticosteroids treatment after IPTW analysis (on time of corticosteroid therapy initiation). Table S7. General characteristics and cause of death of severe patients with COVID-19 and HBV co-infection (16 corticosteroids treatment and 2 non-corticosteroids treatment). Figure S1. Cumulative of overall survival over 28 days between severe patients with corticosteroid therapy and non-corticosteroid after IPTW analysis on the time of corticosteroid therapy initiation. Figure S2. Standardized mean difference before and after IPTW using the variables at the time of corticosteroids initiation. [file 12879_2022_7882_MOESM1_ESM.docx]

**Table S1. The laboratory findings of patients with severe COVID-19 and HBV co-infection on admission. Values are median (IQR) unless stated otherwise.**

| Characteristics | **All patients**  **N=105** | **Survivors**  **N=87** | **Non-survivors**  **N=18** | ***P* value** |
| --- | --- | --- | --- | --- |
| Leukocyte count  (3.5-9.5×10^9^/L) | 5.70 (4.17,8.10) | 5.48 (4.12,7.30) | 9.59 (4.91,15.7) | 0.004 |
| Neutrophil count  (1.8-6.3×10^9^/L) | 3.97 (2.55,6.38) | 3.52 (2.43,5.55) | 7.35 (4.09,13.5) | 0.002 |
| Lymphocyte count  (1.1-3.2×10^9^/L) | 0.97 (0.52, 1.44) | 1.03(0.63, 1.51) | 0.57 (0.46, 0.80) | 0.001 |
| <0.8 (n, %) | 43 (41.0) | 30 (34.5) | 13 (72.2) | 0.003 |
| Platelet count  (125-350×10^9^/L) | 187 (138, 245) | 187 (151, 248) | 173 (92, 229) | 0.190 |
| PT (10.5-13.5s) | 11.9 (11.3,13.1) | 11.8 (11.1, 12.0) | 12.0 (11.4, 13.8) | 0.433 |
| INR (0.8-1.2) | 1.01 (0.96, 1.11) | 1.00(0.95, 1.10) | 1.02 (0.97, 1.15) | 0.505 |
| D-dimer (0-0.5μg/ml) | 0.70 (0.44, 1.85) | 0.60 (0.40, 1.14) | 2.24 (1.12, 6.36) | 0.001 |
| >1 (n, %) | 38 (36.2) | 25 (28.7) | 13 (72.2) | <0.001 |
| ALT (7-40U/L) | 32.5 (21.1, 53.5) | 34.0 (21.0, 50.7) | 30.3 (24.1, 59.8) | 0.738 |
| AST (13-35U/L) | 36.0 (26.0, 48.0) | 35.0 (26.0, 45.0) | 38.4 (29.4, 49.5) | 0.471 |
| ALP (50-135U/L) | 75.0 (57.8, 88.2) | 74.0 (56.3, 83.6) | 77.5 (68.3, 95.7) | 0.116 |
| Bilirubin (0-21μmmol/L) | 12.7 (9.13, 17.6) | 12.3 (8.60, 16.0) | 15.5 (12.3, 20.6) | 0.011 |
| Prealbumin (180-350mg/L) | 101 (60, 140.5) | 98.5 (65.8,160.3) | 101(53,121) | 0.452 |
| Albumin (40-55g/L) | 32.4 (29.1, 36.2) | 33.4 (29.5, 37.4) | 30.1 (27.4, 32.3) | 0.001 |
| < 30 (n, %) | 33 (31.4) | 23 (26.4) | 10 (55.6) | 0.015 |
| Low density lipoprotein  (2.1-3.37mmoL/L) | 2.06 (1.75, 2.56) | 2.03 (1.80, 2.51) | 2.29 (1.61, 2.64) | 0.731 |
| High density lipoprotein  (1.29-1.55mmoL/L) | 1.02 (0.81, 1.16) | 1.03 (0.82, 1.17) | 0.85 (0.75, 1.07) | 0.238 |
| Serum creatinine (41-81μmol/L) | 67.4 (58.8, 80.0) | 67.4 (59.0, 81.3) | 67.6 (57.2, 80.0) | 0.969 |
| High-sensitivity troponin (0-28pg/ml) | 2.90 (1.53, 9.40) | 2.70 (1.71, 7.95) | 4.10 (2.34, 13.0) | 0.242 |
| HS-CRP (0-5mg/L) | 14.0 (6.70, 50.0) | 10.7 (5.93, 45.2) | 50.0 (10.0, 119) | 0.004 |
| IL-6 (0-7pg/ml) | 9.19 (5.99, 12.7) | 8.83 (5.88, 11.5) | 13.6 (8.67, 16.2) | 0.067 |
| Procalcitonin (0-0.05ng/ml) | 0.05 (0.05, 0.10) | 0.05 (0.05,0.07) | 0.10 (0.05, 0.16) | 0.021 |
| LDH (120–250 U/L) | 297 (226, 407) | 289 (216, 375) | 390 (294, 518) | 0.027 |
| ESR (0-20mm/h) | 45.9 (30.0, 65.8) | 45.2 (30.0,62.8) | 49.3 (27.5, 71.6) | 0.951 |
| APACHE II | 5 (4, 8) | 5 (4, 9) | 10 (7, 18) | <0.001 |
| SOFA score | 2 (1,3) | 1 (1, 2) | 3 (2,4) | <0.001 |

COVID-19: coronavirus disease 2019, HBV: Hepatitis B virus, PT: Prothrombin time, INR: International Normalized Ratio, ALT: Alanine amino transferase, AST: Aspartate aminotransferase, ALP: Alkaline phosphatase, HS-CRP: High sensitive c reaction protein, IL-6: interleukin-6, LDH: Lactate dehydrogenase, ESR: Erythrocyte sedimentation rate. APACHE II: Acute Physiology and Chronic Health Evaluation Ⅱ, SOFA: Sequential Organ Failure Assessment.

*P* values indicate differences between survivors and non-survivors. *P* < 0.05 was considered statistically significant.

**Table S2. Characteristics of patients with severe COVID-19 and HBV co-infection on admission according to receiving corticosteroid therapy or not.**

| Characteristics | All patients  N=105 | Corticosteroid  N=55 | Non-corticosteroid  N=50 | | *P value* |
| --- | --- | --- | --- | --- | --- |
| Median age (IQR),yr | 62(54,71) | 62(54,71) | | 62.5(53,71) | 0.766 |
| Gender, n (%) |  |  | |  |  |
| Female | 45(42.9) | 26(47.3) | | 19(38.0) | 0.338 |
| Male | 60(57.1) | 29(52.7) | | 31(62.0) |  |
| Comorbidity |  |  | |  |  |
| Diabetes, n (%) | 16 (15.2) | 9 (16.4) | | 7 (14.0) | 0.736 |
| Hypertension, n (%) | 38 (36.2) | 22 (40.0) | | 16 (32.0) | 0.394 |
| Coronary heart disease, n (%) | 11 (10.5) | 8 (14.5) | | 3 (6.0) | 0.207 |
| COPD, n (%) | 5 (4.8) | 4 (7.3) | | 1 (2.0) | 0.419 |
| Pulmonary tuberculosis, n (%) | 1 (1.0) | 0 (0) | | 1 (2.0) | 0.476 |
| Cancer, n (%) | 9 (8.6) | 3 (5.5) | | 6 (12.0) | 0.397 |
| Cirrhosis, n (%) | 15 (14.3) | 9 (16.4) | | 6 (12.0) | 0.523 |
| HBV infection, n (%) |  |  | |  |  |
| HBeAg positive, n (%) | 5 (4.8) | 2 (3.6) | | 3 (6.0) | 0.913 |
| HBeAb positive, n (%) | 18(17.1) | 12 (21.8) | | 6 (12.0) | 0.182 |
| HBcAb positive, n (%) | 105 (100) | 55 (100) | | 50 (100) | >0.999 |
| Drugs treatment during hospitalization |  |  | |  |  |
| IFN-α, n (%) | 16 (15.2) | 8 (14.5) | | 8 (16.0) | 0.836 |
| Thymosin treatment, n (%) | 29 (27.6) | 18 (32.7) | | 11 (22.0) | 0.220 |
| Immunoglobulin, n (%) | 36 (34.3) | 28 (50.9) | | 8 (16.0) | <0.001 |
| Anti-HBV treatment, n (%) | 9 (8.6) | 5 (9.1) | | 4 (8.0) | 0.842 |
| Anticoagulant therapy, n (%) | 21 (20.0) | 16 (29.1) | | 5 (10.0) | 0.015 |
| Anti-HBV treatment history, n (%) | 8 (7.6) | 5 (9.1) | | 3 (6.0) | 0.820 |

COVID-19: coronavirus disease 2019; HBV: hepatitis B virus, IQR: interquartile range; COPD: chronic obstructive pulmonary disease.

*P* values indicate differences between corticosteroid and non-corticosteroid. *P* **<** 0.05 was considered statistically significant.

**Table S3. The laboratory findings of patients with severe COVID-19 and HBV co-infection on admission according to receiving corticosteroid therapy or not. Values are median (IQR) unless stated otherwise.**

| Characteristics | **All patients**  **N=105** | **Corticosteroid**  **N=55** | **Non-corticosteroid**  **N=50** | ***P* value** |
| --- | --- | --- | --- | --- |
| White blood cell count  (3.5-9.5×10^9^/L) | 5.70 (4.17,8.10) | 5.95 (4.49, 9.52) | 5.18 (3.97, 7.33) | 0.129 |
| Neutrophil count  (1.8-6.3×10^9^/L) | 3.97 (2.55,6.38) | 4.55 (3.09, 8.02) | 3.19 (2.23, 5.35) | 0.013 |
| Lymphocyte count  (1.1-3.2×10^9^/L) | 0.97 (0.52, 1.44) | 0.67 (0.43, 1.11) | 1.21 (0.52, 1.44) | <0.001 |
| <0.8 (n, %) | 43 (41.0) | 31 (56.4) | 12 (24.0) | 0.001 |
| Platelet count  (125-350×10^9^/L) | 187 (138, 245) | 183 (137, 224) | 194 (150, 282) | 0.312 |
| PT (10.5-13.5s) | 11.9 (11.3,13.1) | 11.8 (11.3, 13.4) | 11.9 (11.1, 13.0) | 0.903 |
| INR (0.8-1.2) | 1.01 (0.96, 1.11) | 1.0 (0.96, 1.08) | 1.03 (0.95, 1.14) | 0.831 |
| D-dimer (0-0.5μg/ml) | 0.70 (0.44, 1.85) | 0.89 (0.52, 2.39) | 0.61 (0.46, 1.16) | 0.153 |
| >1 (n, %) | 38 (36.2) | 23 (41.8) | 15 (30.0) | 0.208 |
| ALT (7-40U/L) | 32.5 (21.1, 53.5) | 35 (23, 56) | 30 (20, 48) | 0.446 |
| AST (13-35U/L) | 36.0 (26.0, 48.0) | 38 (29, 50) | 32 (24.7, 46) | 0.235 |
| ALP (50-135U/L) | 75.0 (57.8, 88.2) | 75 (61, 98) | 74 (57, 82) | 0.366 |
| Bilirubin (0-21μmmol/L) | 12.7 (9.13, 17.6) | 12.5 (9.48, 18.3) | 13.2 (8.95, 16.9) | 0.820 |
| Prealbumin (180-350mg/L) | 101 (60, 140.5) | 96 (53, 121) | 110 (71, 202) | 0.087 |
| Albumin (40-55g/L) | 32.4 (29.1, 36.2) | 31.9 (28.2, 35.0) | 33.5(30.1, 37.1) | 0.161 |
| < 30 (n, %) | 33 (31.4) | 20 (36.4) | 13 (26.0) | 0.253 |
| High density lipoprotein  (1.29-1.55mmoL/L) | 1.02 (0.81, 1.16) | 1.03 (0.81, 1.25) | 0.99 (0.79, 1.08) | 0.488 |
| Serum creatinine (41-81μmol/L) | 67.4 (58.8, 80.0) | 68.7 (53.8, 82.6) | 64.0 (60.2, 76.4) | 0.769 |
| High-sensitivity troponin (0-28pg/ml) | 2.90 (1.53, 9.40) | 6.0 (1.8, 12.9) | 3.55 (1.80, 11.1) | 0.836 |
| Hs-CRP (0-5mg/L) | 14.0 (6.70, 50.0) | 48.5 (26.3, 94.9) | 18.2 (4.8, 79.8) | 0.015 |
| IL-6 (0-7pg/ml) | 9.19 (5.99, 12.7) | 11.2 (7.3, 14.5) | 9.15 (6.54, 11.49) | 0.244 |
| Procalcitonin (0-0.05ng/ml) | 0.05 (0.05, 0.10) | 0.06 (0.05, 0.16) | 0.05 (0.05, 0.05) | 0.003 |
| LDH (120–250 U/L) | 297 (226, 407) | 370 (285,515) | 271 (205, 332) | 0.001 |
| ESR (0-20mm/h) | 45.9 (30.0, 65.8) | 50.0 (30.0,72.0) | 43.5 (30.0, 58.9) | 0.421 |
| APACHE II | 5 (4, 8) | 6(4,8) | 5 (4, 9) | 0.387 |
| SOFA score | 2 (1,3) | 2 (1,3) | 2 (1,3) | 0.832 |

COVID-19: coronavirus disease 2019, HBV: hepatitis B virus, PT: prothrombin time, INR: International Normalized Ratio, ALT: Alanine amino transferase, AST: Aspartate aminotransferase, ALP: Alkaline phosphatase, Hs-CRP: High sensitive c reaction protein, IL-6: interleukin-6, LDH: Lactate dehydrogenase, ESR: Erythrocyte sedimentation rate, APACHE II: Acute Physiology and Chronic Health Evaluation Ⅱ, SOFA: Sequential Organ Failure Assessment.

*P* values indicate differences between corticosteroid and non-corticosteroid. *P* **<** 0.05 was considered statistically significant.

**Table S4 The laboratory findings of patients with severe COVID-19 and HBV co-infection according to receiving corticosteroid therapy or not on the time of corticosteroid therapy initiation. Values are median (IQR) unless stated otherwise.**

| Characteristics | **All patients**  **N=105** | **Corticosteroid**  **N=55** | **Non-corticosteroid**  **N=50** | ***P* value** |
| --- | --- | --- | --- | --- |
| White blood cell count  (3.5-9.5×10^9^/L) | 5.67 (4.16,7.40) | 6.94 (4.62, 9.30) | 4.51 (3.73, 6.59) | 0.001 |
| Neutrophil count  (1.8-6.3×10^9^/L) | 3.66 (2.70,5.78) | 4.99 (3.24, 7.46) | 2.93 (2.22, 4.70) | <0.001 |
| Lymphocyte count  (1.1-3.2×10^9^/L) | 0.97 (0.52, 1.51) | 0.78 (0.45, 1.51) | 1.07 (0.80, 1.51) | 0.109 |
| <0.8 (n, %) | 40 (38.1) | 28 (50.9) | 12 (24.0) | 0.005 |
| Platelet count  (125-350×10^9^/L) | 186 (141, 238) | 186 (138, 235) | 181 (141, 242) | 0.918 |
| PT (10.5-13.5s) | 11.7 (11.4,12.7) | 12.5 (11.5, 12.8) | 11.7 (11.2, 11.7) | 0.014 |
| INR (0.8-1.2) | 1.00 (0.97, 1.08) | 1.08 (0.98, 1.10) | 1.00 (0.96, 1.00) | 0.011 |
| D-dimer (0-0.5μg/ml) | 1.00 (0.53, 2.28) | 1.00 (0.60, 2.67) | 0.83 (0.52, 1.80) | 0.285 |
| >1 (n, %) | 45 (42.9) | 25 (45.5) | 20 (40.0) | 0.573 |
| ALT (7-40U/L) | 32.0 (19.9, 48.0) | 33 (23, 50) | 29.75 (16, 45.80) | 0.131 |
| AST (13-35U/L) | 34.1 (23.0, 45.0) | 38.7 (30, 48) | 31 (22, 43.9) | 0.121 |
| ALP (50-135U/L) | 74.0 (64.0, 83.0) | 72 (57, 88) | 74 (68, 82) | 0.592 |
| Bilirubin (0-21μmmol/L) | 12.0 (8.60, 17.1) | 12.0 (8.5, 17.3) | 12.65 (8.6, 17.1) | 0.608 |
| Albumin (40-55g/L) | 32.5 (29.0, 36.2) | 32.1 (28.8, 35.1) | 33.4(29.1, 36.9) | 0.294 |
| < 30 (n, %) | 34 (32.4) | 18 (32.7) | 16 (32.0) | 0.937 |
| Serum creatinine (41-81μmol/L) | 67.9 (57.9, 79.0) | 68.6 (54.0, 79.3) | 67.7 (59.3, 76.0) | 0.684 |
| High-sensitivity troponin (0-28pg/ml) | 2.00 (0.04, 7.60) | 4.05 (0.03, 11.50) | 1.80 (0.30, 3.60) | 0.225 |
| HS-CRP (0-5mg/L) | 27.2 (5.88, 50.9) | 48.5 (15.5, 66.6) | 6.2 (3.0, 34.5) | <0.001 |
| IL-6 (0-7pg/ml) | 9.98 (7.85, 10.21) | 10.21 (8.06, 10.21) | 9.98 (7.80, 9.98) | 0.077 |
| Procalcitonin (0-0.05ng/ml) | 0.05 (0.05, 0.07) | 0.06 (0.05, 0.10) | 0.05 (0.05, 0.05) | 0.001 |
| APACHE II | 8 (6, 10.5) | 7(6,10) | 9 (7, 11) | 0.038 |
| SOFA score | 5 (5,7) | 5 (5,6) | 5.5 (5,7) | 0.344 |

COVID-19: coronavirus disease 2019, HBV: hepatitis B virus, PT: prothrombin time, INR: International Normalized Ratio, ALT: Alanine amino transferase, AST: Aspartate aminotransferase, ALP: Alkaline phosphatase, Hs-CRP: High sensitive c reaction protein, IL-6: interleukin-6, LDH: Lactate dehydrogenase, ESR: Erythrocyte sedimentation rate, APACHE II: Acute Physiology and Chronic Health Evaluation Ⅱ, SOFA: Sequential Organ Failure Assessment.

P values indicate differences between corticosteroid and non-corticosteroid. P < 0.05 was considered statistically significant.

**Table S5. Logistic regression modeling evaluating risk factors associated with 28-day mortality in patients with severe COVID-19 and HBV co-infection (on the time of corticosteroid therapy initiation).**

| Variables | Univariate logistic regression | | | Multivariate logistic regression | | |  |
| --- | --- | --- | --- | --- | --- | --- | --- |
|  | **OR** | **95%CI** | ***P* value** | **OR** | **95%CI** | ***P* value** | |
| Age | 1.060 | 1.006-1.118 | 0.029 | 1.068 | 1.008-1.132 | 0.027 |  |
| Gender (Male vs Female) | 1.796 | 0.576-5.597 | 0.313 |  |  |  |  |
| Comorbidity | 2.933 | 0.879-9.792 | 0.080 |  |  |  |  |
| Lymphocyte count < 0.8 ×10^9^/L | 9.951 | 2.621-37.784 | 0.001 | 7.599 | 1.847-31.261 | 0.005 |  |
| D-dimer >1 μg/ml | 2.571 | 0.858-7.708 | 0.092 |  |  |  |  |
| ALT > 40 u/L | 0.850 | 0.271-2.669 | 0.781 |  |  |  |  |
| Albumin < 30 g/L | 2.423 | 0.822-7.144 | 0.109 |  |  |  |  |
| Hs-CRP >5 mg/L | 12.354 | 0.673-226.763 | 0.094 |  |  |  |  |
| Corticosteroid treatment | 8.195 | 1.758-38.191 | 0.007 | 7.126 | 1.352-37.561 | 0.021 |  |

Comorbidity: Other underlying diseases excluding HBV infection. ALT: alanine amino transferase. Hs-CRP: High sensitive c reaction protein.

*P* values indicate differences between corticosteroid and non-corticosteroid. *P* **<** 0.05 was considered statistically significant. OR: odds ratio, CI: confidence intervals.

**Table S6. The primary and secondary outcomes of patients with severe COVID-19 and HBV co-infection between corticosteroids and non-corticosteroids treatment after IPTW analysis (on time of corticosteroid therapy initiation).**

| Outcome | OR | 95%CI | *P* value |
| --- | --- | --- | --- |
| Primary Outcomes |  |  |  |
| 28-day mortality | 7.279 | 2.286-23.173 | 0.001 |
| In-hospital mortality | 8.462 | 2.679-26.728 | <0.001 |
| Secondary Outcomes |  |  |  |
| Acute liver injury | 1.846 | 1.065-3.197 | 0.029 |
| Acute kidney injury | 0.659 | 0.305-1.423 | 0.288 |
| Acute cardiac injury | 1.491 | 0.696-3.192 | 0.304 |
| Invasive mechanical ventilation | 0.685 | 0.328-1.432 | 0.314 |
| CRRT | 0.165 | 0.027-1.022 | 0.053 |
| SARS-CoV-2 RNA positive more than 20d | 5.989 | 3.183-11.269 | <0.001 |
| Sepsis shock | 0.403 | 0.211-0.769 | 0.006 |
| ARDS |  |  | >0.999 |

COVID-19: coronavirus disease 2019, HBV: hepatitis B virus, ARDS: acute respiratory distress syndrome, CRRT: continuous renal replacement therapy.

*P* values indicate differences between corticosteroid and non-corticosteroid. *P* < 0.05 was considered statistically significant. OR: odds ratio, CI: confidence intervals.

**Table S7. General characteristics and cause of death of severe patients with COVID-19 and HBV co-infection (16 corticosteroids treatment and 2 non-corticosteroids treatment).**

| ID | Gender | | Age | | Time from symptom onset to admission, d | | | Time from symptom onset to death, d | Highest  D-dimer  (0-0.5μg/ml) | Cause of death |
| --- | --- | --- | --- | --- | --- | --- | --- | --- | --- | --- |
| 1 | Male | | 46 | | 7 | | | 19 | 52.68 | Severe ARDS, acute cardiac injury, acute liver injury, cardiac arrest. |
| 2 | Female | | 51 | | 6 | | | 30 | 21.14 | Severe ARDS, DIC |
| 3 | Male | | 55 | | 10 | | | 15 | 2.06 | Severe ARDS, acute cardiac injury, liver injury. |
| 4 | Male | | 56 | | 14 | | | 22 | 21.04 | Severe ARDS, liver injury. |
| 5 | Male | | 57 | | 7 | | | 13 | 70.86 | Severe ARDS，liver failure, carcinoma of nasopharynx, type 2 diabetes. |
| 6 | Female | | 61 | | 9 | | | 69 | 22.24 | Severe ARDS, sepsis shock, liver injury, acute kidney injury, DIC, primary hypertension. |
| 7 | Female | | 61 | | 16 | | | 25 | 11.39 | Severe ARDS, liver injury, cardiac arrest, colorectal carcinoma. |
| 8 | Female | | 71 | | 10 | | | 23 | 80.00 | Cerebral hemorrhage, moderate ARDS, primary hypertension, type 2 diabetes. |
| 9 | Male | | 73 | | 8 | | | 19 | 34.44 | Severe ARDS, sepsis shock, liver injury, acute kidney injury, acute cardiac injury. |
| 10 | Male | 75 | | 9 | | | 13 | | 53.44 | Severe ARDS, liver injury. |
| 11 | Female | | 79 | 7 | | | 30 | | 10.44 | Severe ARDS, liver injury, primary hypertension, type 2 diabetes, coronary heart disease. |
| 12 | Male | | 83 | 3 | | | 22 | | 12.30 | Severe ARDS, primary hypertension, coronary heart disease, COPD |
| 13 | Male | | 75 | 12 | | | 25 | | 4.36 | Severe ARDS, sepsis shock, liver injury, acute kidney injury, acute cardiac injury, primary hypertension, type 2 diabetes. |
| 14 | Male | | 70 | 16 | | 50 | | | 4.81 | Severe ARDS, DIC, massive alimentary hemorrhage, liver injury, acute cardiac injury. |
| 15 | Male | | 71 | 11 | | 22 | | | 22.00 | Severe ARDS, sepsis shock, liver injury, acute kidney injury, acute cardiac injury, primary hypertension. |
| 16 | Female | | 68 | 21 | | 36 | | | 31.26 | Severe ARDS, DIC, massive alimentary hemorrhage. |
| 17* | Male | | 81 | 30 | | 50 | | | 0.40 | Severe ARDS, DIC, primary hypertension, type 2 diabetes. |
| 18* | Male | | 81 | 30 | | 40 | | | 1.69 | Severe ARDS, DIC, septic shock, acute cardiac injury, prostate cancer. |

COVID-19: coronavirus disease 2019, HBV: hepatitis B virus, ARDS: acute respiratory distress syndrome, DIC: disseminated intravascular coagulation, COPD: chronic obstructive pulmonary disease**.**

* Non-corticosteroids treatment.

**Figure S1: Cumulative of overall survival over 28 days between severe patients with corticosteroid therapy and non-corticosteroid after IPTW analysis on the time of corticosteroid therapy initiation.**


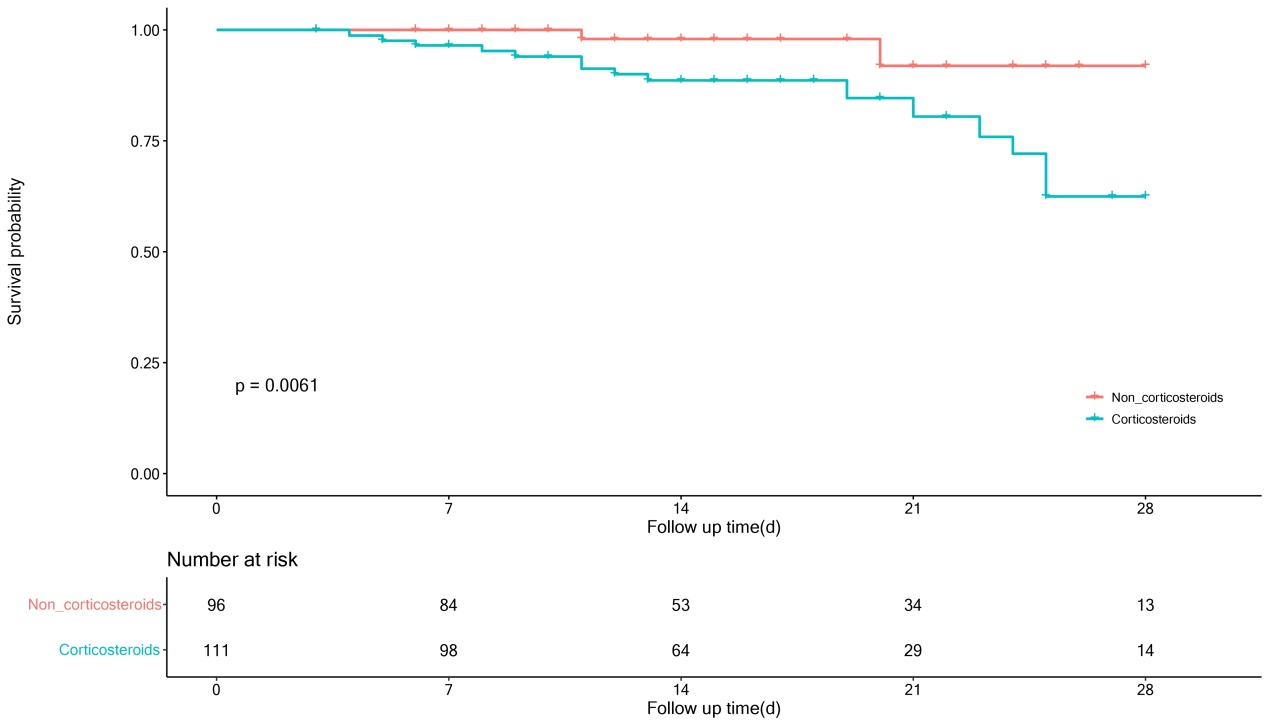


**Figure S2 Standardized Mean Difference before and after IPTW using the variables at the time of corticosteroids initiation.**


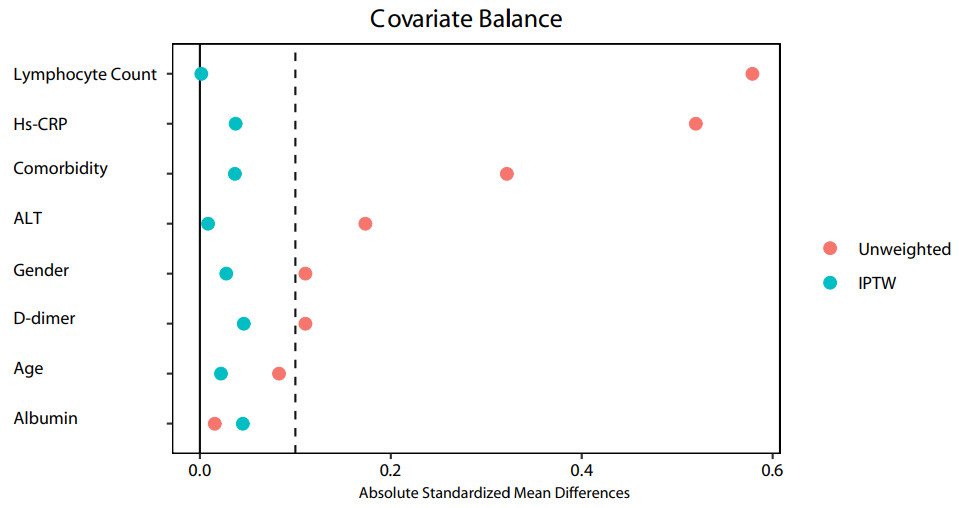


Hs-CRP, High sensitive c reaction protein, ALT: Alanine amino transferase.

**Definitions**

HBsAg-positive, Hepatitis B virus e antigen (HBeAg)-positive, Hepatitis B virus c antibody-positive were defined as HBsAg >0.5 IU/ml, HBeAb >1 IU/ml, HBcAb >2 IU/ml, respectively. HBV DNA-positive was defined as higher than 3log10 copies/ml. Diagnosis of cirrhosis was based on liver function tests, ultrasounds and CT screening. Sequential organ failure assessment score (SOFA) and acute physiology and chronic health evaluation scoring system II score (APACHE II) were computed to standardize predicted mortality.

The Berlin Definition was used to define ARDS^1^. Severe or critical SARS-CoV-2 pneumonia is characterized by bilateral multifocal pulmonary infiltrates that result in acute hypoxemic respiratory failure, often fulfilling the Berlin criteria for diagnosis of ARDS. These include severe arterial hypoxemia despite supplemental oxygen and bilateral infiltrates on chest imaging that are not solely attributable to cardiac dysfunction^2^. AKI was diagnosed according to the KDIGO criteria^3^. Septic shock was based on the Third International Consensus Definition for Sepsis and Septic Shock. Acute liver injury was defined as the peak values of serum alanine aminotransferase (ALT) ≥1 × the upper limit of normal (ULN): Acute liver injury was categorized as mil-moderate if ALT was > ULN but < five times ULN, and severe if ALT was > five times ULN^4^. Acute cardiac injury was defined by serum levels of specific cardiac biomarkers (e.g. high-sensitive cardiac troponin I) were above the 99th percentile upper reference limit by each local laboratory based on age and gender and/or new abnormalities were identified in electrocardiography and echocardiography^5^. The time of COVID-19 onset was defined according to the time of initial symptoms as self-reported by patients. Identification of SARS-CoV-2 RNA was based on RT-PCR testing. The virus clearance criteria were defined as two consecutive negative results of PCR detection at 24 hours interval of. The clearance date was considered as the time of the first test.

References

1. Force ADT, Ranieri VM, Rubenfeld GD, et al. Acute respiratory distress syndrome: the Berlin Definition. JAMA. 2012; 307(23): 2526-33.

2. Ferrando C, et al. Clinical features, ventilatory management, and outcome of ARDS caused by COVID-19 are similar to other causes of ARDS. Intensive Care Med. 2020;46(12):2200–2211.

3. Khwaja A. KDIGO clinical practice guidelines for acute kidney injury. Nephron Clin Pract. 2012; 120(4): c179-84.

4. Phipps MM, Barraza LH, LaSota ED, et al. Acute Liver Injury in COVID-19: Prevalence and Association with Clinical Outcomes in a Large U.S. Cohort. Hepatology. 2020; 72(3): 807-817.

5. Wang P, Sha J, Meng M, et al. Risk factors for severe COVID-19 in middle-aged patients without comorbidities: a multicenter retrospective study. J Transl Med. 2020; 18(1): 461.
